# The effectiveness of chest T-tube drainage in uniportal video-assisted thoracic surgery

**DOI:** 10.1093/icvts/ivad114

**Published:** 2023-07-20

**Authors:** Alexandro Patirelis, Federica Carlea, Sara Lo Torto, Federico Tacconi, Vincenzo Ambrogi

**Affiliations:** Department of Thoracic Surgery, Tor Vergata University Polyclinic, Rome, Italy; Department of Thoracic Surgery, Tor Vergata University Polyclinic, Rome, Italy; Department of Thoracic Surgery, Tor Vergata University Polyclinic, Rome, Italy; Department of Thoracic Surgery, Tor Vergata University Polyclinic, Rome, Italy; Department of Thoracic Surgery, Tor Vergata University Polyclinic, Rome, Italy

**Keywords:** T-tube, Chest drainage, Tube kinking, Uniportal video-assisted thoracic surgery, Postoperative pneumothorax, Thoracic surgery

## Abstract

**OBJECTIVES:**

Uniportal incision located at 4th or 5th intercostal space represents a problem for the correct drainage of distal areas of pleural cavity. The T-shaped tube can drain both the extremities of pleural space. In this study, we evaluated the effectiveness of T-chest tube compared to classic chest tube after uniportal video-assisted thoracic surgery.

**METHODS:**

We compared the effectiveness of T-tube and classic 28 CH chest drainage after different surgical procedures in uniportal video-assisted thoracic surgery: lobectomies, wedge resections and pleural and mediastinal biopsies. As primary end points, drained effusion and evidence of pneumothorax at postoperative day 1, subcutaneous emphysema, tube kinking, obstruction and necessity of repositioning or postoperative thoracentesis were considered. Pain at 6 and 24 h after surgery, pain at tube removal and mean hospitalization were analysed as secondary end points.

**RESULTS:**

A total of 109 patients were selected for the study, 51 included to the T-tube group while the other 58 ones to the control group with classic drainage. Patients with T-tube showed a significantly lower rate of pneumothorax (29.4% vs 63.8%; *P* < 0.001), tube kinking (5.9% vs 27.6%; *P* = 0.003) and need of repositioning (2.0% vs 12.1%; *P* = 0.043). No significant results were obtained in subcutaneous emphysema (*P* = 0.26), tube obstruction (*P* = 0.32), drained effusion (*P* = 0.11) and need of postoperative thoracentesis (*P* = 0.18). Patients with T-tube complained of <6 h after surgery (*P* < 0.001). Conversely, T-tube removal was reported to be more painful (*P* < 0.001).

**CONCLUSIONS:**

Chest T-tube can achieve significantly lower rate of postoperative pneumothorax, kinking and repositioning with less pain 6 hours after surgery compared to classic tube.

**Figure:**
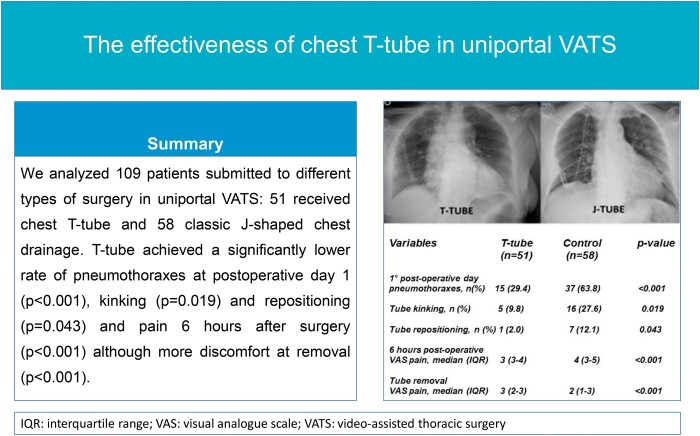

## INTRODUCTION

The widespread use of uniportal video-assisted thoracic surgery (VATS) brought many changes in surgical techniques but also in the chest tube positioning [1]. Indeed, this approach allowed a shift from double tube placement to single drainage.

Given the level of the single incision in uniportal VATS, usually located at 4th or 5th intercostal space, the tube can properly drain either the air located in the apex of the pleural space or the fluids accumulated in the bases. To avoid this incomplete drainage, the chest tube could be adapted with a J shape, with a proximal curve descending towards the base and ascending to the apex. However, this setting could be highly jeopardized to kinking. Conversely, the creation of a second port to specifically allocate a more caudal drainage would be contrary to the philosophy of the procedure.

**Figure 1:**
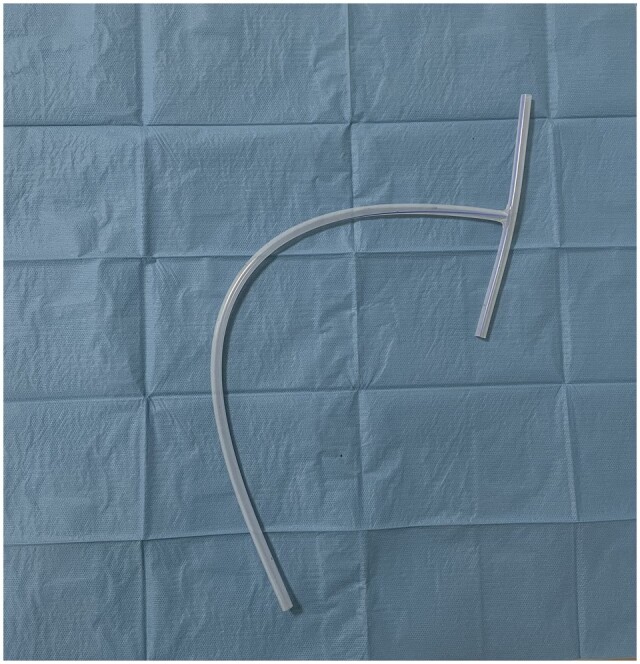
Chest T-tube, developed on purpose by Redax SpA (Poggio Rusco, Italy).

**Figure 2:**
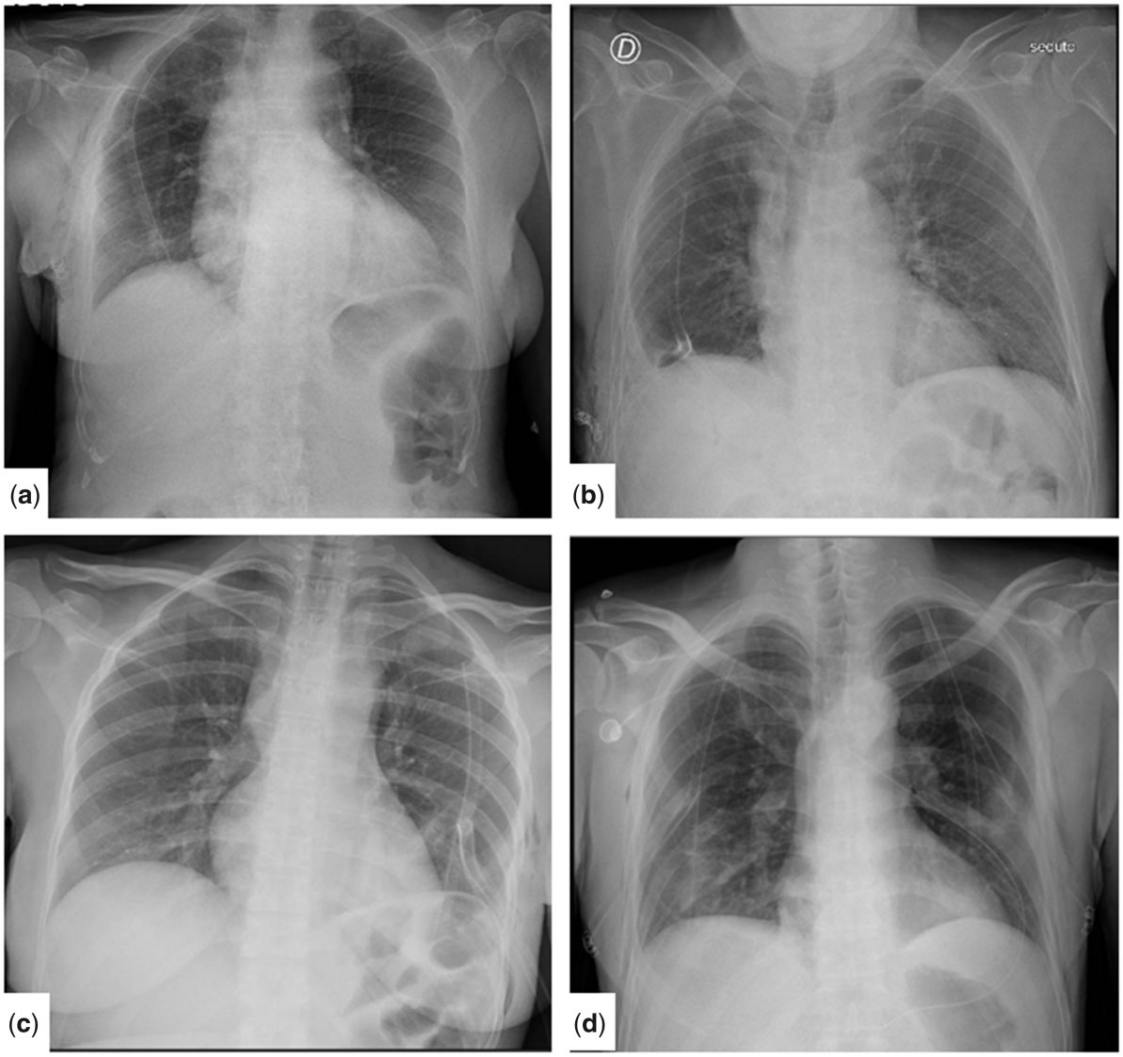
Examples of use of T-tube after uniportal video-assisted thoracic surgery on X-ray. (**a**) Right wedge resection; (**b**) right lower lobectomy; (**c**) left mediastinal biopsy; and (**d**) bilateral lung metastasectomy (wedge resections).

To overcome this issue, our group started to employ off-label a T-shaped tube (Figure 1), usually applied in biliary duct surgery [2] and developed on purpose by Redax SpA (Poggio Rusco, Italy), as a pleural drainage [3] (Figure 2) after having obtained the authorization from the Internal Review Board of our institution.

In this prospective non-randomized study, we compared the effectiveness of T-tube after uniportal VATS to a control group drained with a classic 28 CH chest tube.

## MATERIALS AND METHODS

### Ethical statement

This study was approved by Tor Vergata Polyclinic Internal Review Board (approval n. 12.21), which did not deem a separate written patient informed consent for the use of chest T-tube to be compulsory. Every patient was aware about the possibility of using a chest T-tube drainage and released a fully informed and signed consent within our standard agreement for surgery as an explained footnote. The authorization for the use of personal data was included in the form signed by the patient.

### Patient selection

From July 2020 to May 2022, we evaluated the effectiveness of 2 different types of chest drainage in a group of patients submitted to different surgical procedures in uniportal VATS, with a single utility port at 4th or 5th intercostal space. Procedures were lobectomy, wedge resection, pleural and mediastinal biopsy. Lobectomies were performed for pulmonary cancers whereas wedge resections were carried out for suspected pulmonary nodules revealed to be benign at frozen section histological examination or for pulmonary metastasectomy. At the end of surgery, a T-tube or a classic chest tube was placed to drain air and pleural fluids. Patients with pleural effusion associated with likely inexpandable lung and patients with interstitial disease were ruled out from both groups to avoid bias on postoperative pneumothorax rate.

T-tube was a 24 CH tube developed on purpose for thoracic uniportal surgery. Classic chest tube was a 28 CH drainage modelled in a J-shape to simultaneously reach the apex and the base of the hemithorax.

The allocation within each group was mainly determined according to the habit of the 2 different teams performing uniportal VATS in our institution: one of them preferred T-tube while the other one classic chest tube. However, sometimes the allocation between groups depended also on other unpredictable factors, such as the availability of the tube in the operating room, regardless the equipe’s preference.

All the patients underwent a chest X-ray during the first postoperative day to evaluate tube position and pulmonary re-expansion. Pain was expressed by the study population at fixed intervals (6 and 24 h after surgery and during tube removal) through visual analogue scale (VAS) (0 = absent; 10 = worst pain ever) [4].

Data about drained effusion during the first postoperative day and further postoperative procedures, such as thoracentesis, and days of hospitalization were collected by clinical records.

### Objectives

The aim of this prospective non-randomized study was to compare the effectiveness of 2 different types of chest tube after uniportal VATS. As primary end-points, we considered evidence of pneumothorax at chest X-ray performed at the first postoperative day (present vs absent), the onset of clinically significant subcutaneous emphysema, episodes of tube kinking or obstruction of the tube, need of chest tube repositioning, drained effusion during first postoperative day in ml and inadequate pleural liquid drainage implying the necessity of postoperative thoracocentesis. For this last end point, we considered the presence of at least 1200 ml of pleural effusion deemed at ultrasound evaluation by a certified operator (Federica Carlea) as cut-off for thoracentesis. As secondary end points, we assessed differences of perceived postoperative pain at fixed intervals and mean length of hospitalization.

### Statistics

Statistical analysis was performed through SPSS (IBM Corp. Released 2016. IBM SPSS Statistics, Version 26.0; Armonk, NY, USA: IBM Corp.). *P*-value <0.05 was considered statistically significant.

Descriptive analysis of data was executed using Mann–Whitney non-parametric test for continuous variables and Pearson’s χ-square for categorical ones.

To evaluate possible predictors of complications a binary logistic regression was performed including the following variables: type of tube, sex, age (dichotomized according to the median, 66 years old), type of surgery (dichotomized in lobectomies versus other surgeries), side of surgery and the presence of emphysema.

No propensity score matching was applied given the small analysed sample.

## RESULTS

A total of 109 patients submitted to different surgical procedures in uniportal VATS were included in this study, with a single utility port at 4th or 5th intercostal space. Surgical procedures were lobectomy (*n* = 51), wedge resection (*n* = 32), pleural (*n* = 14) and mediastinal biopsy (*n* = 12). At the end of surgery, a T-tube was placed in 51 patients (51/109; 46.8%) while in the other 58 patients (58/109; 53.2%), a classic chest tube was used to drain air and pleural fluids. Demographic and clinical features of the study group are reported in Table 1.

**Table 1:** Demographic and clinical features of the study group

Variables	T-tube group (*n* = 51)	Control group (*n* = 58)	*P*-Value
Age (years), median (IQR)	66 (61–68)	67 (61–71)	0.15
Gender, *n* (%)			0.66
Male	26 (51.0%)	32 (55.2%)	
Female	25 (49.0%)	26 (44.8%)	
Presence of emphysema, *n* (%)			0.51
Yes	18 (35.3%)	24 (41.4%)	
No	33 (64.7%)	34 (58.6%)	
Type of surgery, *n* (%)			0.63
Lobectomy	27 (52.9%)	24 (41.4%)	
Wedge resection	14 (27.5%)	18 (31.0%)	
Pleural biopsy	5 (9.8%)	9 (15.5%)	
Mediastinal biopsy	5 (9.8%)	7 (12.1%)	
Side of surgery, *n* (%)			0.38
Right	28 (54.9%)	27 (46.6%)	
Left	23 (45.1%)	31 (53.4%)	
Right side surgery, *n* (%)			
Lobectomy	17 (63.0%)	11 (45.8%)	0.22
Wedge resection	6 (42.9%)	7 (38.9%)	0.82
Pleural biopsy	2 (40.0%)	5 (55.6%)	0.58
Mediastinal biopsy	3 (60.0%)	4 (57.1%)	0.92

IQR: interquartile range.

### Primary end points

Results regarding primary end points are reported in Table 2. A statistically significant between-group difference was found in the evidence of pneumothorax during the first postoperative day at chest X-ray, with less cases observed in the T-tube group (15/51, 29.4% vs 37/58, 63.8%; *P* < 0.001).

**Table 2:** Results of primary and secondary end points in the study group

Variables	T-tube group (*n* = 51)	Control group (*n* = 58)	*P*-Value
Primary end points			
1st POD pneumothoraxes, *n* (%)			**<0.001**
Present	15 (29.4%)	37 (63.8%)	
Absent	36 (70.6%)	21 (36.2%)	
Subcutaneous emphysema, *n* (%)			0.26
Present	5 (9.8%)	10 (17.2%)	
Absent	46 (90.2%)	48 (82.8%)	
Tube kinking, *n* (%)			**0.019**
Yes	5 (9.8%)	16 (27.6%)	
No	46 (90.2%)	42 (72.4%)	
Tube obstruction, *n* (%)			0.32
Yes	4 (7.8%)	8 (13.8%)	
No	47 (92.2%)	50 (86.2%)	
Tube repositioning, *n* (%)			**0.043**
Yes	1 (2.0%)	7 (12.1%)	
No	50 (98.0%)	51 (87.9%)	
1st POD drained effusion (ml), median (IQR)	220 (150–320)	175 (130–290)	0.11
Postoperative thoracentesis, *n* (%)			0.18
Yes	0 (0.0%)	2 (3.4%)	
No	51 (100.0%)	56 (96.6%)	
Secondary end points			
6 h postoperative VAS pain median (IQR)	3 (3–4)	4 (3–5)	**<0.001**
24 h postoperative VAS pain median (IQR)	3 (2–4)	3 (2–4)	0.35
Tube removal VAS pain median (IQR)	3 (2–5)	2 (1–3)	**<0.001**
Hospitalization (days) median (IQR)	3 (2–3)	3 (2–4)	0.54

IQR: interquartile range; POD: postoperative day; VAS: visual analogue scale. Significant P-values (<0.05) are bold.

Another significant difference was observed in the episodes of tube kinking, with a greater number of cases in the control group (16/58, 27.6% vs 3/51, 5.9% of the T-tube group; *P* = 0.003). Only in 3 cases of the T-tube group kinking was present at chest X-ray. Despite kinking the T-tube remained functional in almost all these events and repositioning was necessary just once (1/51, 2.0% vs 7/58, 12.1% in the control group; *P* = 0.043).

No statistically significant differences were found in the number of subcutaneous emphysema (*P* = 0.26), tube obstruction (*P* = 0.32), drained effusion during first postoperative day (*P* = 0.11) or in postoperative thoracentesis (*P* = 0.18).

We also performed a binary logistic regression to evaluate possible predictors of complications (the finding of pneumothorax, subcutaneous emphysema, tube kinking, obstruction or need of repositioning and/or need of postoperative thoracentesis). As a result, the presence of J-shaped classic chest tube revealed to be a predictor of complication, with an odds ratio (OR) of 4.71 [95% confidence interval (CI), 1.91–11.62, *P* = 0.001]. Other predictors of complications were the presence of emphysema (OR = 2.83, 95% CI, 1.11–7.20, *P* = 0.029) and lobectomy compared to other surgeries (OR = 0.37, 95% CI, 0.15–0.95, *P* = 0.038).

A subgroup analysis of primary end points of patients undergoing lobectomy was carried out. Indeed, these patients, according to the results of the binary logistic regression and to the greater complexity of surgery, were more exposed to possible complications. This analysis confirmed a greater significant incidence of pneumothoraxes during the first postoperative day in the control group (19/24 vs 10/27 cases, *P* = 0.002) as in the cases of tube kinking (10/24 vs 3/27 cases, *P* = 0.012).

### Secondary end points

As depicted in Table 2, a statistically significant difference was detected in pain estimated 6 h after surgery, revealing one-point difference of the median VAS in the presence of the T-tube {3 [interquartile range (IQR) 3–4] vs 4 (IQR 3–5); *P* < 0.001}. No statistically significant difference was found comparing pain of the 2 groups the day after surgery (*P* = 0.35). At tube removal, T-tube was related to a more painful experience compared to the control group [3 (IQR 2–5) vs 2 (IQR 1–3) at median VAS; *P* < 0.001]. No difference was found in mean length of hospitalization (*P* = 0.54).

## DISCUSSION

Uniportal VATS has become increasingly popular in recent years [1]. According to the majority of the studies, this approach seems to offer a faster recovery and shorter hospitalization length as well as reduction in postoperative pain and complications [5–7].

In accordance with the trend of reducing the impact of surgery, also the attitude of chest drainage has meaningfully changed. Even in the case of major operations the habit of positioning 2 chest drain has progressively abandoned with the transition to a single chest tube routine usage [8]. Furthermore, some authors have even suggested the possibility to avoid chest tube placement after minor surgery and in selected patients [9, 10].

Unfortunately, the level of uniportal VATS approach can represent by itself an obstacle to the correct chest drain positioning [11]. Indeed, through the single incision at the mid-thorax level routinely used in uniportal VATS surgery, a single tube cannot at once reach both the apex and the base. On the other hand, the creation of an additional port would trespass the peculiar trait of the procedure. In order to overcome this obstacle, the tube could be inserted following a J-shaped pathway, with a proximal convexity at basal level and the distal part facing towards the pleural dome. Nevertheless, this position is associated with a higher risk of tube angulation and kinking, requiring chest-tube relocation, as shown in this study. Moreover, the achievement of an ideal J shape is not always easy to finalize and also depends on the experience and the skills of the surgeon [8].

The T-tube was originally developed by the German surgeon Hans Kehr (1862–1916) [12] to stent biliary ducts allowing bile external drainage. Another type of T-tube is the Montgomery, used in otolaryngology in patients with tracheostomy to prevent tracheal stenosis [13]. T-tube developed on purpose for thoracic surgery and used in this study, with its opposite branches can reach both extremities of the hemithorax despite mid-thorax insertion without tricky manoeuvres or tortuous trips. Patients with T-tube presented less evidence of pneumothorax at the first postoperative day without increasing the necessity of thoracocentesis for evacuating pleural effusion. This is probably due to branch length (9 cm each one with a global widespan of >18 cm), thus resulting adequate for almost all hemithorax size, even in the slenderest subjects. The episodes of clinically significant subcutaneous emphysema in T-tube group were likely due to upward climbing of the superior branch of the T-tube, thus touching the pleural dome and plugging of the only existing higher hole. These complications were promptly resolved by slightly retracting the T-tube.

**Figure 3:**
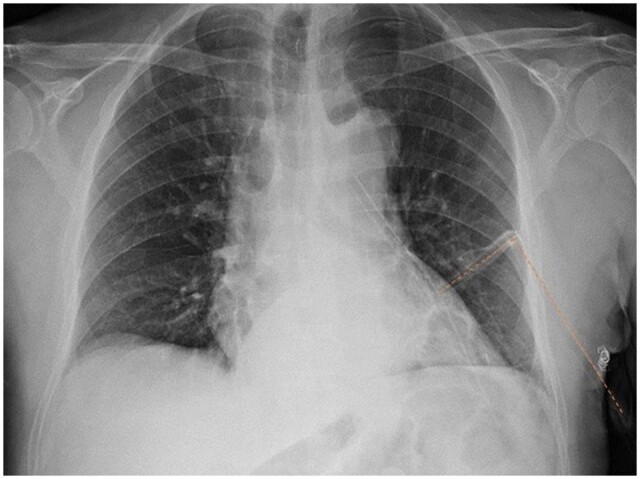
Example of T-tube kinking on chest X-ray (highlighted with dotted line).

In addition, the risk of kinking was significantly reduced in T-tube group. We explained this finding with the easier positioning set and the absence of angulations required compared to J-shape tube. Indeed, the T-tube is already preformed and, once having occupied the mid-hemithorax position, maintains by itself diametrically opposed extremities. In the rare event of T-tube kinking, due to its displacement following pulmonary re-expansion (Fig. 3), the drainage remained functional in almost all these occasions.

After the first observational study, some concerns about the possibility of drain collapse according to Bernoulli’s effect were raised, stating that the confluence of the 2 branches inside a single horizontal portion might create a negative pression [14]. As a matter of fact, this pression fluctuation was measured as minimal when compared to the variation induced by standard breathing [15]. Furthermore, the study demonstrated that this different orientation of the 2 branches did not influence the passage of liquids throughout the tube as proved by the equivalent number of tube obstruction in the 2 groups.

The T-tube has a smaller size than the classic 28 CH chest tube used by our group, thus resulting in less pain complained by the patients during postoperative recovery. The use of a larger calibre in the control group was necessary to allow adequate drainage despite the possibility of kinking. Although better tolerability, patients with T-tube complained more discomfort during removal. This act resulted more awkward than the removal of a straight traditional chest tube since the 2 branches have to slip through a relatively small skin incision, hence causing more pain. As little trick, we found that tube retrieval can be made easier with a minimal rotation during removal to make the 2 branches twist around themselves.

### Limitations

Indeed, we have to acknowledge that this study has several limitations. First of all, this was a non-randomized study and the allocation between group criteria was mainly based on the habit of the 2 operating teams. Nevertheless, the 2 equips worked in the same institution, with the same assistants, and performed the surgical operations with the same technique and skill after analogue training. The only difference was the preference of the tube.

In addition, due to the relatively limited sample size up till now accrued, no propensity score matching has yet been feasible. To overcome these problems, a perspective controlled randomized trial would be strongly desirable and will be set within a short time.

Moreover, the analysis of potential confounding factors in the logistic regression was restricted only to the ones available from the database which we considered as the most predictive of postoperative complications.

## CONCLUSION

The progression in surgical techniques often originates unexpected problems producing new challenges. Proposed solutions can often be quite brainy but sometimes they are very intuitive and simple. This can be the case of a well-functioning chest drainage in uniportal thoracic surgery. Indeed, the presence of a single incision at mid-thorax can cause some problems of chest tube orientation. According to our study chest T-tube can achieve significantly lower rate of postoperative pneumothorax, kinking and repositioning with less pain compared to classic tube. The T-tube could represent an easy yet effective solution to classic chest drainage even in major surgery. A proper randomized prospective study will be necessary to finally validate the effectiveness of T-tube in uniportal VATS.

## Data Availability

The data underlying this article will be shared on reasonable request to the corresponding author.

## Notes

Presented at the XXI Società Italiana di Endoscopia Toracica (SIET) National Meeting, Rome, Italy, 29 September–1 October 2022.

## ABBREVIATIONS

CI: Confidence interval
IQR: Interquartile range
OR: Odds ratio
VAS: Visual analogue scale
VATS: Video-assisted thoracic surgery
